# Editorial: Allergic diseases through precision medicine

**DOI:** 10.3389/falgy.2026.1842074

**Published:** 2026-04-17

**Authors:** Patricia Agudelo-Romero, Enza D'Auria, Anthony Bosco

**Affiliations:** 1 Wal-yan Respiratory Research Centre, The Kids Research Institute Australia, Perth, WA, Australia; 2 School of Molecular Sciences, The University of Western Australia, Perth, WA, Australia; 3 European Virus Bioinformatics Centre, Friedrich-Schiller-Universitat Jena, Jena, Germany; 4 Department of Pediatrics, Buzzi Children’s Hospital, Milan, Italy; 5 Department of Biomedical and Clinical Sciences, University of Milan, Milan, Italy; 6 Asthma and Airway Disease Research Center, University of Arizona, Tucson, AZ, United States; 7 Department of Immunobiology, The University of Arizona College of Medicine, Tucson, AZ, United States

**Keywords:** allergic diseases, asthma, atopic dermatitis, climate change, food allergy, omics, precision medicine, rhinitis

Allergic diseases are rising worldwide, especially in childhood, and their clinical diversity increasingly exposes the limits of traditional phenotype-based classifications (1–4). Genetic susceptibility, environmental exposures, epithelial barrier biology, and immune pathways interact to shape highly variable disease trajectories and treatment responses (1, 3, 4). In this context, precision medicine is no longer only an aspirational concept, but a practical effort to define meaningful endotypes, identify clinically useful biomarkers, and connect biological insight to prevention and care (3, 4).

**Figure 1:**
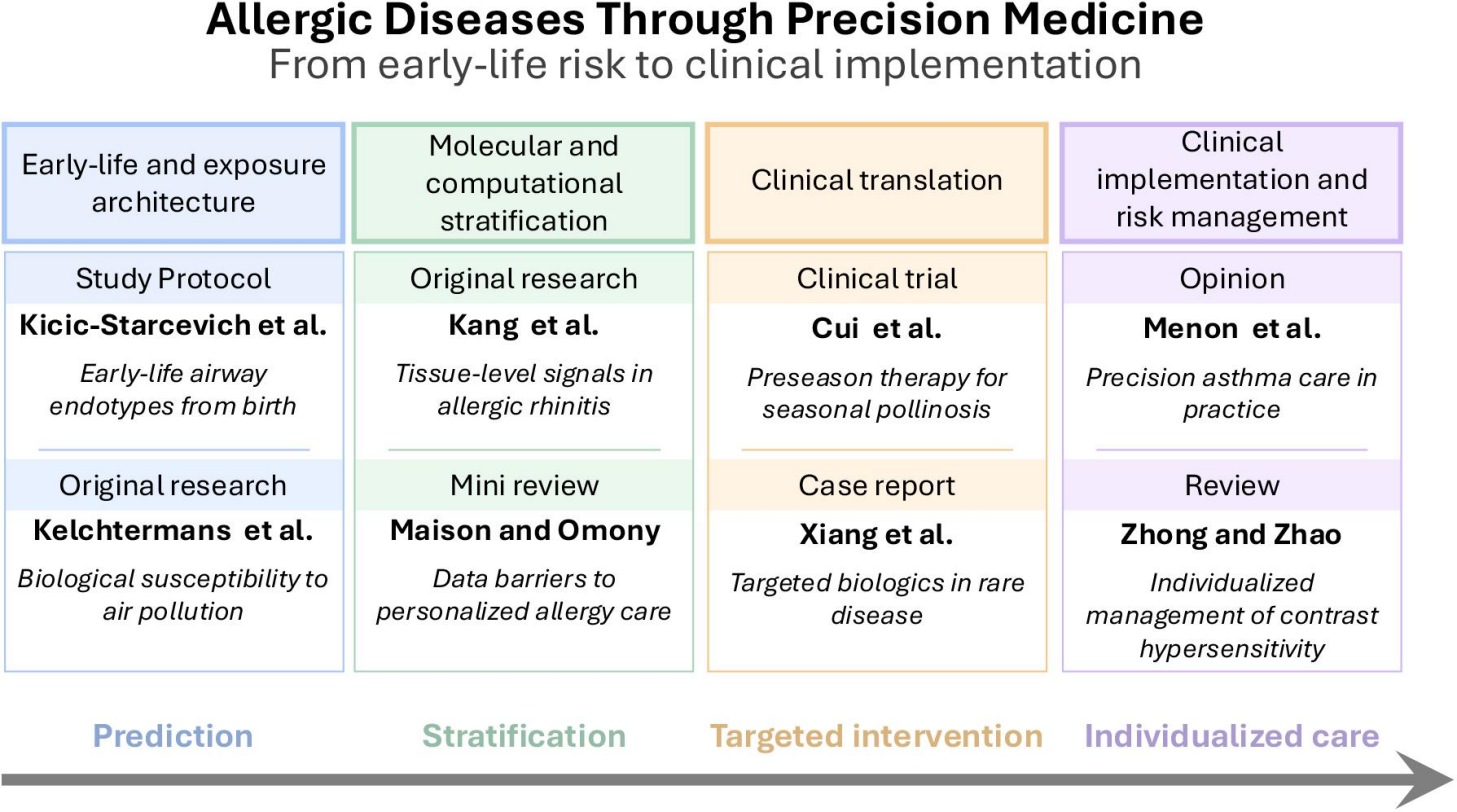
Conceptual overview of the research topic “*Allergic diseases through precision medicine*”: the contributions in this topic span a continuum from early-life risk, through molecular and computational stratification, to clinical translation, as well as clinical implementation and risk management. Together, they illustrate that precision medicine in allergy is not a single approach but a layered framework integrating developmental, mechanistic, and clinical perspectives.

The eight contributions in this Topic trace that effort across four connected fronts (Figure 1). Early-life risk mapping is represented by Kicic-Starcevich et al., who describe the AERIAL birth cohort, which combines multi-omics approaches with app-based symptom monitoring to capture viral exposures and identify epithelial endotypes linked to wheeze and asthma from birth through early childhood. Kelchtermans et al. extend this perspective by examining gene-environment interactions in pediatric asthma, suggesting that susceptibility to air pollution may differ across biologically distinct subgroups.

Molecular and computational stratification is addressed through complementary mechanistic and analytical perspectives. Kang et al. report increased *SOCS3* and *Eotaxin* expression in nasal tissue from patients with allergic rhinitis, contributing tissue-level molecular evidence relevant to disease mechanisms. Maison and Omony, in turn, highlight the computational barriers that continue to slow translation, including heterogeneous phenotypes, incomplete biomarker validation, and fragmented clinical data.

Clinical translation is illustrated by Cui et al., whose randomized trial in Artemisia pollinosis shows that preseason prophylaxis can reduce seasonal nasal symptoms, suggesting that precision care may also involve optimizing the timing of established therapies. Xiang et al. describe Netherton syndrome in the context of genetic diagnosis and pathway-targeted biologic therapy, illustrating the potential of tailored treatment in rare and highly selected patients, while also underscoring the limited evidentiary weight of case reports.

Clinical implementation and risk management are addressed by Menon et al., who argue that precision medicine must complement, rather than replace, the foundations of good asthma care, including accurate diagnosis, inhaler technique, adherence, and corticosteroid stewardship. Zhong and Zhao broaden the scope further by reviewing hypersensitivity to iodinated contrast media, emphasizing that individualized risk assessment and management also matter in settings where mechanisms remain incompletely understood.

Together, these papers show that precision medicine in allergy is developing across several fronts at once, but not at the same pace. In some areas, it is already shaping clinical choices; in others, it remains mainly mechanistic or exploratory. The next step is not simply to generate more mechanistic insight, but to determine when it can inform clinically meaningful decisions in prevention, diagnosis, or treatment.
